# Valoración de un cambio de protocolo del cribado prenatal mediante la inclusión del diagnóstico prenatal no invasivo

**DOI:** 10.1515/almed-2019-0020

**Published:** 2020-04-07

**Authors:** Rocío Cabra-Rodríguez, Guadalupe Bueno Rodríguez, Cristina Santos Rosa, Miguel Ángel Castaño López, Sonia Delgado Muñoz, Antonio León-Justel

**Affiliations:** UGC de Análisis Clínicos, Hospital Juan Ramón Jiménez, Huelva, Spain; Servicio de Análisis Clínicos, Hospital Infanta Elena, Huelva, Spain; Hospital Juan Ramón Jiménez, Ronda Norte s/n, 21005, Huelva, Spain

**Keywords:** aneuploidias, diagnóstico prenatal, no invasivo

## Abstract

**Objetivos:**

El diagnóstico prenatal no invasivo (DPNI) es un test que permite detectar en la sangre materna las principales alteraciones cromosómicas del feto durante el embarazo. El objetivo es evaluar el DPNI, valorando su rendimiento dentro del Programa Andaluz de Cribado de Anomalías Congénitas del Sistema Sanitario Público Andaluz.

**Métodos:**

Estudio observacional retrospectivo en el que se analizan los DPNI realizados desde su incorporación, además del número de procedimientos diagnósticos invasivos tras la implementación del DPNI en gestantes incluidas en el programa entre marzo del año 2016 y agosto del 2017.

**Resultados:**

Se realizaron 6.258 cribados combinados en gestantes de primer y segundo trimestre, con una cobertura de la población del 95%; se obtuvo un cálculo de riesgo elevado (≥1/280) en 250 gestantes, de los cuales el DPNI se aplicó en 200 gestantes después de asumir las pérdidas. La sensibilidad obtenida fue de 100% (IC 95%:76,84 a 100%) y la especificidad de 99,46% (IC 95%:97,04 a 99,99%).

**Conclusiones:**

Este test es muy sensible, con alta especificidad. En nuestro estudio, la incorporación del DPNI en la práctica clínica minimiza las pérdidas fetales y reduce en un 70% la realización de procedimientos invasivos.

## Introducción

La Medicina Fetal se enfrenta en la actualidad a grandes retos, entre los que se encuentra la detección prenatal de las anomalías genéticas que genera en el individuo grandes discapacidades. Por estos motivos se ha promovido el desarrollo de diversas estrategias de cribado poblacional en pacientes gestantes mediante pruebas inocuas y de procesamiento sencillo para clasificar aquellas gestantes con riesgo elevado de presentar alguna anomalía genética en sus fetos [1].

En la década de los años 70, el cribado prenatal de las cromosomopatías fetales más frecuentes dependía exclusivamente de la edad de la madre. Posteriormente, en los años 80 se desarrollaron marcadores bioquímicos (alfafetoproteína y fracción beta de la gonadotropina coriónica humana [β-hCG] total o libre) que conjuntamente con la valoración de la edad materna, permitía detectar los fetos con síndrome de Down (trisomías 21[T21]) aunque de manera poco eficaz. Con el paso de los años se incorporaron nuevos marcadores bioquímicos (proteína plasmática asociada al embarazo [PAPP-A]) y ecográficos (translucencia nucal [TN]), permitiendo incrementar notablemente las tasas de detección hasta el 85–90% para las trisomías 21 [2].

En el año 2009 se hizo una propuesta de cribado prenatal de cromosomopatías para todo el Sistema Sanitario Público de Andalucía (SSPA). Dicha propuesta adoptó las recomendaciones de la Sociedad Española de Ginecología y Obstetricia (SEGO) e incluyó el cribado combinado del primer trimestre (CC1°T) dentro de un programa más amplio denominado Programa Andaluz para el Cribado Prenatal de Anomalías Congénitas (PACAC) [3, 4].

Tras la implantación del PACAC, se desarrolla en el Sistema Público Andaluz de Salud una herramienta informática corporativa denominada siPACAC, que permite efectuar en la actualidad los cálculos necesarios para establecer el riesgo de la gestación, tanto en el primer trimestre (PAPP-A, β-hCG libre y TN antes de la 14^a^ semana de gestación), como en el segundo trimestre (β-hCG libre y alfafetoproteína) de T21 y otras trisomías [2, 3].

Los datos son analizados con la aplicación informática denominada siPACAC, la cual, proporciona un cálculo del riesgo. Si el riesgo es elevado, se recomienda a las pacientes la realización de un procedimiento invasivo, como la amniocentesis o la biopsia corial, con los riesgos que ello supone para la madre y el feto [4], [5].

La posibilidad de que un recién nacido presente algún tipo de defecto congénito es de un 2–3% al nacimiento, de éstos un 1–1,5% se deben a malformaciones (60% del total) y un 0,5–1% (12–15% del total) se deben a cromosomopatías [6].

Para el diagnóstico de confirmación de estas anomalías cromosómicas, tanto en el primer como en el segundo trimestre, cuando existe un cribado de riesgo elevado, se requiere la obtención de material genético a través de procedimientos invasivos como la biopsia corial o la amniocentesis, procedimientos diagnósticos que no son inocuos y que conllevan un riesgo asociado de pérdidas fetales (aproximadamente del 1%). Además, supone un coste económico y emocional añadido para la futura madre. En estos casos siempre es necesario el consentimiento informado de la paciente, tanto para poder realizar procedimientos invasivos como para la revocación voluntaria [4]. Las pruebas de detección de anomalías cromosómicas más utilizadas son el cariotipo convencional y el estudio mediante FISH o QF-PCR de los cromosomas 13, 18, 21, X e Y. A diferencia del cariotipo tradicional, la FISH o la QF-PCR no requieren un cultivo celular previo de origen fetal, posibilitando la obtención de resultados en 48 horas [7].

Recientemente se han desarrollado nuevas técnicas de biología molecular como apoyo a este cribado de aneuploidías en pacientes gestantes. Hablamos por tanto del diagnóstico prenatal no invasivo (DPNI) o también NIPD, del inglés "Non Invasive Prenatal Diagnosis", que hace referencia a un test de ADN fetal en sangre materna que permite el estudio de las características genéticas del feto sin acceso directo al útero, y por lo tanto, sin riesgo de pérdida fetal asociada [8].

Con este test se detectan en la sangre materna las principales alteraciones cromosómicas del feto durante el embarazo: cromosomas 21 (Síndrome de Down), 18 (Síndrome de Edwards), 13 (Síndrome de Patau) y X (Síndrome de Turner). Para el síndrome de Down la sensibilidad es del 99% y la tasa de falsos positivos es menor del 0,1% [9].

El test se lleva a cabo a través de un simple análisis de sangre de la madre. No implica riesgo ni para la madre ni para el feto, siendo considerada de alta eficacia diagnóstica [10], [11].

El análisis de ADN fetal en sangre materna para la detección de trisomías 21, 18 y 13 es un método de detección efectivo, pero costoso, disponible desde la 10^a^ semana de gestación. Una estrategia para maximizar el rendimiento a un coste reducido es ofrecer este test supeditado al resultado del cribado combinado del primer trimestre que se utiliza actualmente [11], [12].

La proporción de ADN placentario (fracción fetal) estudiada en este análisis de ADN fetal en sangre materna es un factor determinante para la valoración del test y debe ser tenida en cuenta a la hora de la interpretación de los resultados. De forma general, la literatura existente en la actualidad marca un punto de corte de una fracción fetal mínima del 4% para emitir un resultado fiable, y si es < 4% limita la sensibilidad y especificidad del test [13], [14]. En DPNI no debemos obviar que se trata de un método de cribado cuyos resultados positivos requieren confirmación mediante técnica invasiva y los resultados negativos no excluyen la condición al 100% [15], [16], [17], [18].

El objetivo principal de este trabajo es la evaluación del nuevo test de diagnóstico prenatal no invasivo (Harmony®), calculando la sensibilidad, especificidad y tasa de falsos positivos en una población seleccionada previamente con cribado de alto riesgo en el Programa Andaluz de Cribado de Anomalías Congénitas.

## Materiales y métodos

Es un estudio observacional retrospectivo en el que analizamos el número de tests de DPNI realizados, desde la incorporación en la cartera de servicios de un hospital del SSPA, en nuestra área sanitaria. Además, se determina el número de procedimientos diagnósticos invasivos (amniocentesis y biopsia corial) tras la implementación del DPNI en las gestantes incluidas en el programa PACAC desde marzo del año 2016 a agosto del 2017 en el Área Hospitalaria Juan Ramón Jiménez. Se reclutaron las pacientes hasta el mes de agosto del 2017, no ampliándose el periodo hasta la fecha actual, ya que se tuvo que comprobar que las gestaciones habían llegado a término. Se revisaron además, a todos los recién nacidos tras el parto, con CC1°-2°T con resultado de alto riesgo para aneuploidías.

Este test prenatal no invasivo (Harmony®) se ha incorporado en nuestro hospital como un paso previo en aquellos casos de presentar un riesgo elevado con el CC1°-2° T que precisan confirmación con la prueba invasiva (amniocentesis o la biopsia corial).

**Figura 1:**
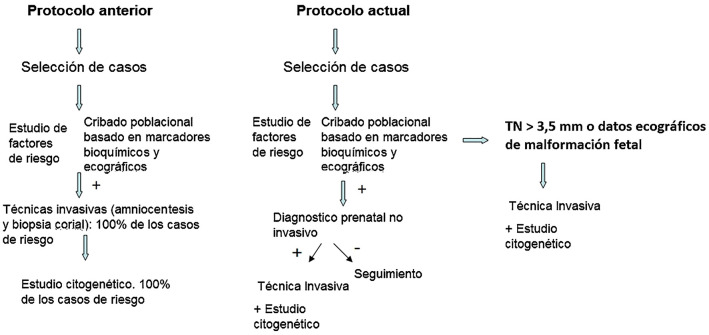
Estrategia diagnóstica con la incorporación del Diagnóstico Prenatal No Invasivo en nuestro centro hospitalario.

El DPNI se oferta a las gestantes con un cribado de aneuploidias del primer o segundo trimestre con riesgo elevado, ≥1/280 en el caso de trisomía 21 y ≥1/150 para trisomía 13 y trisomía 18, como un paso intermedio antes de realizar el procedimiento invasivo. En el caso que el DPNI sea de bajo riesgo (<1/10.000=0,01%) se realiza el seguimiento habitual. Si el DPNI es de alto riesgo se le propone a la gestante la posibilidad de realizar un procedimiento invasivo para confirmar la alteración detectada con estudio citogenético (cariotipo y citoarray en el caso de que proceda) (Figura 1).

El DPNI (Harmony®) se realizó mediante extracción de sangre total, enviándose a un laboratorio externo Megalab, previo consentimiento informado de la paciente. En aquellos casos en los que la fracción fetal no era la adecuada (fracción fetal < 4%) se completó con una nueva determinación y nueva extracción de sangre. El resultado de dicho test estaba disponible en el plazo de 7 a 9 días laborables.

Este test se basa en el análisis del ADN libre total circulante (ADN-lc) en el plasma materno del que aproximadamente un 10% (3–13%) es de origen placentario (no exclusivamente fetal) [12]. Para la realización del cálculo de riesgos para las diferentes aneuploidías se estudia el *pool* total de ADN de origen materno y placentario mediante diversas técnicas (recuento relativo por microarrays dirigidos o genotipado) para calcular el riesgo final [13], [19].

Para calcular la sensibilidad, especificidad y la tasa de falsos positivos se ha realizado un seguimiento durante 25 meses a las gestantes en las que se aplicó el DPNI con resultado de bajo riesgo (<1/10.000=0.01%). Valoramos a todos los recién nacidos tras el parto y comprobamos que estos no presentaban ningún rasgo dismórfico o alteración fenotípica al nacimiento ni al alta hospitalaria mediante la revisión de la historia clínica.

Los casos de alto riesgo de sospecha de aneuploidías o >99% con DPNI fueron confirmados con cariotipo y/o citoarray del líquido amniótico. Por el contrario, en las gestantes en las que el DPNI fue de bajo riesgo con datos ecográficos normales se continuaron los controles habituales, revisando todas las historias clínicas, la hoja de obstetricia y la exploración de los recién nacidos en el momento del parto y al alta hospitalaria. Se registró el número de revocaciones, tanto para el DPNI como para los procedimientos invasivos, además de las interrupciones voluntarias de embarazo justificadas únicamente por las malformaciones ecográficas halladas sin otros estudios complementarios. Los casos de aborto espontáneo o diferido y las interrupciones voluntarias por malformación fetal fueron consideradas como pérdidas del estudio y no se contabilizaron para el análisis estadístico. Además, otras pérdidas registradas para el cálculo de la sensibilidad y especificidad, fueron las de aquellas pacientes respecto a las que no tuvimos información de sus recién nacidos por haber sido atendidas en otros centros privados y las revocaciones del DPNI.

Los datos demográficos de las pacientes y el cálculo de riesgo del cribado del primer y segundo trimestre para aneuploidias fueron extraídos del programa siPACAC.

Los resultados del DPNI (Harmony™) y otros estudios citogenéticos fueron recogidos a través de programa Infinity® Roche Diagnostics y de las historias clínicas de las pacientes.

Por último, para el análisis estadístico de los datos se utilizó el programa informático MedCalc® Easy-to-use statistical software,versión 11.0.

## Resultados

**Figura 2:**
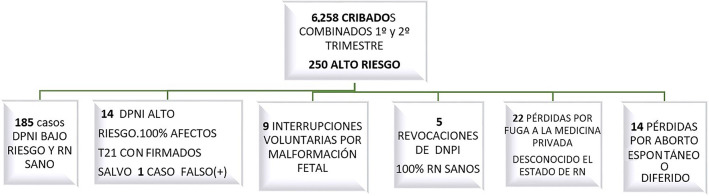
Resultados tras la implementación del Diagnóstico Prenatal No Invasivo en nuestra Área Sanitaria.

Se realizaron 6.258 cribados combinados en las gestantes de primer y segundo trimestre (CC1°-2°T), con una cobertura de la población del 95%; se obtuvo un cálculo de riesgo elevado (≥1/280) en 250 gestantes. En el primer trimestre se detectaron 211 cribados de riesgo elevado y 39 en el segundo. Se encontró solo un falso negativo con el cribado combinado de aneuploidias en el primer trimestre al que no se le realizó el DPNI. Se estimó la sensibilidad y especificidad del DPNI solo en los casos en que el CC1°-2°T para T21 asociado a T13/T18 o ambos, eran de un riesgo elevado. Finalmente, el número de casos estudiados fue de 200, no habiéndose incluido para este cálculo los casos de pérdidas de pacientes que continuaron el seguimiento del embarazo en la medicina privada y en los que, por tanto, no se tuvo acceso a la exploración del recién nacido. Además, tampoco se computaron los casos de abortos espontáneos o voluntarios y revocaciones para el DPNI (Figura 2).

Todos los DPNI de alto riesgo (>99%) ocurrieron en gestaciones con feto único para T21 y se confirmaron todos los resultados con cariotipo, exceptuando un caso, que hubo que completar el estudio con citoarrays de líquido amniótico. No se registró ningún caso de revocación para procedimiento invasivo tras un DPNI de alto riesgo.

**Figura 3:**
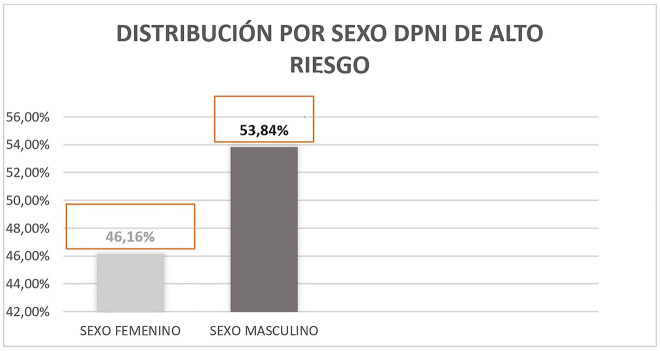
Distribución por sexo del diagnóstico prenatal no invasivo >99% de riesgo para trisomía 21.

Entre los datos sociodemográficos y datos clínicos de las pacientes destacan los siguientes: edad media materna de 36,06 años (18–44), gestantes con hábito tabáquico 30%, técnicas de reproducción asistida 4%, pacientes con diabetes mellitus 1,5% y peso materno medio en la primera visita de control de la gestación 67,3 kg (41–120). El resto de resultados se exponen en la Tabla 1 y Figura 3.

**Tabla 1:** Rendimiento del Diagnóstico Prenatal No Invasivo para trisomía 21 y trisomía 21 asociado a síndrome de Patau / síndrome de Edwards.

Variable	Cribado 1 y 2 trimestre
Número de pacientes	6.258 DPNI en 200 Riesgo elevado
Verdaderos positivos	14
Verdaderos negativos	185
Falsos positivos	1
Falsos negativos	0
Sensibilidad (intervalo confianza 95%)	100% (76,84% a 100%)
Especificidad (intervalo confianza 95%)	99,46% (97,04% a 99,99%)
Valor predictivo positivo (intervalo confianza 95%)	93,33% (68,05% a 99,83%)
Valor predictivo negativo (intervalo confianza 95%)	100% (98,03% a 100%)
Prevalencia de enfermedad	7% (3,88% a 11,47%)
Tasa de falsos positivos	0,53%

De las 9 interrupciones voluntarias del embarazo, en dos de ellas se realizó, previa a la interrupción, la amniocentesis del líquido amniótico para su posterior estudio por PCR, cariotipo y citoarrays, con el fin de catalogar las alteraciones ecográficas encontradas. En las restantes no se realizó el procedimiento invasivo ya que las malformaciones eran de gravedad e incompatibles con la vida, originando que las gestantes decidieran finalizar el embarazo. Un caso fue confirmado de síndrome de Down y el otro no se llegó al diagnóstico pese a repetirse la prueba en dos ocasiones y ante la persistencia de alteraciones ecográficas de tipo hidrops fetal se solicitó estudio de secuenciación masiva, confirmando el diagnóstico de síndrome de Noonan.

Se realizaron un total de 18 amniocentesis durante el periodo de estudio, las dos mencionadas en el párrafo anterior y las 16 indicadas por ser el DPNI de alto riesgo, con objeto de realizar la confirmación. Al revisar los procedimientos invasivos realizados en el mismo periodo estudiado del año anterior, previo a la incorporación del DPNI (marzo 2014 a agosto 2015), se habían realizado en 60 casos los procedimientos invasivos. Por lo tanto, si comparamos ambos periodos se reducen en un 70% la realización de procedimientos invasivos. Para finalizar, destacamos que la tasa de revocación de DPNI en nuestro estudio fue de un 2,85%.

## Discusión

Los resultados obtenidos en nuestro estudio muestran una sensibilidad y especificidad muy elevadas (99,46% y 100% respectivamente).

Se observa un valor predictivo negativo y positivo elevados (100 y 93,33% respectivamente), por lo que puede considerarse un excelente test para la detección de dichas cromosomopatías, trisomía 21 fundamentalmente. Este método también analiza los cromosomas 18 y 13 y tiene el potencial de llegar a cubrir todo el genoma [20], [21].

Los resultados obtenidos en nuestro trabajo, tanto la tasa de falsos positivos del 1% como el valor predictivo positivo del 93,33% (IC95%: 68,05–99,83%), fueron algo mejores que los descritos por Peral Camacho et al. [2], los cuales estudiaron un grupo de 6.584 mujeres y obtuvieron una tasa de falsos positivos del 3,2%. Por otra parte, nuestros resultados fueron similares a los obtenidos por otros autores como Norton et al. [9], que estudiaron 15.841 mujeres y obtuvieron una tasa de falsos positivos del 0,06% y un valor predictivo positivo del 80,9% (IC95%: 66,7–90,9%).

La sensibilidad y especificidad es menor en el caso de las gestaciones múltiples, pero en nuestro estudio todos los DPNI fueron realizados en embarazos con feto único, por lo que no hubo que excluir ningún caso [14].

Entre las limitaciones de nuestro trabajo, tenemos que destacar sobre todo las pérdidas importantes del estudio debido a que en algunas pacientes fue imposible obtener la información necesaria para realizar el seguimiento del embarazo por causas diversas como: seguimiento en clínicas privadas, abortos espontáneos o diferidos e interrupciones voluntarias del embarazo por malformaciones ecográficas, de las que no se pudo confirmar dichas alteraciones cromosómicas con el estudio de los restos abortivos.

Este estudio no fue diseñado para la comparación del CC1°-2°T y DPNI, por lo que este último no se realizó sobre la población total de estudio (n=6.258), sino que solo se realizó en aquellos casos seleccionados de CC1°-2°T de alto riesgo (n=200), tras descontar 50 pacientes computadas como pérdidas del estudio. Por ello, creemos importante realizar un mayor número de investigaciones, donde el número de embarazos con cromosomopatías confirmados, fuese mayor para poder extrapolar el resultado a otras poblaciones.

En conclusión, la incorporación del DPNI reduce de forma importante la realización de procedimientos invasivos según nuestro estudio, además se minimizan las pérdidas fetales y el estrés psicológico, mejorando la práctica clínica diaria [11], [22]. Sin embargo, el DPNI presenta como inconveniente su alto coste y su escasa disponibilidad que hace que no esté al alcance de muchos laboratorios [23]. Para muchos países, esto a menudo requiere el transporte internacional de la muestra pudiendo repercutir en el resultado de la prueba debido a una demora en la fase preanalítica [8], [24].

Siguiendo las recomendaciones de los Informes de Evaluación de Tecnologías Sanitarias (SESCS) respecto al análisis de ADN fetal en sangre materna para la detección de las trisomías 21, 18 y 13, queda limitada como prueba de cribado prenatal contingente o de segunda línea, a las gestantes en las que previamente se haya establecido un riesgo alto de trisomía fetal en los cromosomas T21, T18 o T13 con el CC1°-2°T [25].

Es importante establecer protocolos consensuados que definan los criterios de indicación de la prueba en todo el Sistema Nacional de Salud, que incluyan; la definición de riesgo alto a partir del cual se recomienda su uso, circunstancias en las que se desaconseja la prueba, limitación de la técnica y el consejo genético [26], [27].
